# Mpox Vaccination Willingness, Concern Profiles, and Associated Factors Among Men Who Have Sex with Men in Changsha, China

**DOI:** 10.3390/vaccines14050428

**Published:** 2026-05-10

**Authors:** Yingying Zhou, Wenqiang Wang, Yun Kuang, Qiang Hu, Lin Shen, Qiangming Xie, Zhi Xie

**Affiliations:** 1 Changsha Center for Disease Control and Prevention, Changsha 410004, China; 2 Changsha Zonda-Sunshine Social Work Center, Changsha 410000, China; cszdyg@catchangsha.cn

**Keywords:** mpox vaccination, men who have sex with men, vaccination willingness, respondent-driven sampling, integrated prevention

## Abstract

**Background**: Mpox vaccination is an important prevention strategy for men who have sex with men (MSM), yet responses to vaccination may not be adequately captured by a simple willing-versus-unwilling framing. We examined correlates of vaccination willingness and heterogeneity within the delayed/refused responses subgroup in Changsha, China. **Methods**: A cross-sectional survey was conducted using respondent-driven sampling (RDS). Vaccination willingness was classified as immediate willingness versus delayed/refused responses. Analyses included cluster-robust logistic regression, RDS-weighted regression, generalized estimating equations, and a recruiter-linked network-lag model. Among respondents with delayed/refused responses, concern profiles were explored using unsupervised clustering of standardized concern items. **Results**: Among 405 recruited MSM without a self-reported mpox infection history, immediate willingness and delayed/refused responses were nearly equally distributed, indicating that lack of immediate willingness was common. Across primary models, ever use of pre-exposure prophylaxis (PrEP) and higher mpox-related information exposure frequency were the most consistent correlates of immediate willingness versus delayed/refused responses, whereas basic sociodemographic variables showed little evidence of independent association. Within the delayed/refused group, three partially overlapping patterns emerged: broadly elevated cross-domain concern, low-concern delay with few strongly endorsed barriers, and more selective safety- and burden-related concerns. These findings suggest that a lack of immediate willingness may arise through different psychosocial pathways rather than a single common mechanism. **Conclusions**: Mpox vaccination willingness among MSM in Changsha appeared to be shaped more by prevention-related behaviors and psychosocial factors than by basic sociodemographic profiles alone. Vaccination strategies may benefit from cross-topic sexual-health communication, integrated prevention efforts, and subgroup-sensitive approaches to delayed or refused willingness.

## 1. Introduction

The emergence of the multi-country mpox outbreak in 2022 and its rapid international spread drew widespread public health attention. Although the acute phase of the outbreak has subsided, mpox remains a disease of continuing public health relevance and is still subject to international surveillance and response [1], particularly as more recent reports have documented ongoing local transmission through sexual networks of men who have sex with men (MSM) in several European countries [2]. During the 2022 outbreak, transmission was concentrated disproportionately among MSM, underscoring their priority for prevention and control [3]. Mpox is a zoonotic disease caused by the monkeypox virus (MPXV) and can be transmitted through close physical contact, contaminated materials, and intimate or sexual contact [3,4]. Although most mpox infections are self-limiting, therapeutic options remain limited, and prevention and public health measures, therefore, remain central to outbreak control [5,6].

Vaccination is one of the most cost-effective strategies for controlling emerging infectious diseases [7,8]. For mpox, currently available vaccines such as ACAM2000 and JYNNEOS have provided preventive options in some settings [5], while vaccine development and implementation remain more limited in others, including China [9,10]. However, the public health impact of vaccination depends not only on vaccine availability but also on willingness to be vaccinated [11,12]. Vaccine hesitancy remains a major barrier to immunization and may be particularly relevant in the context of mpox, where concerns about adverse effects, uncertainty about vaccine effectiveness, stigma, low perceived susceptibility, and access-related barriers may all shape vaccination willingness [13,14]. Understanding willingness to receive mpox vaccination is, therefore, important not only for estimating potential coverage but also for informing targeted communication and delivery strategies in populations at elevated risk [15].

This issue is especially important among MSM. In addition to facing stigma, privacy concerns, and limited conventional sampling frames in survey research [16], MSM also constitute a population in which vaccination willingness may be shaped by a broader psychosocial, informational, and behavioral context. This includes prior awareness of mpox, perceived transmission risk, vaccine beliefs, patterns of information exposure, and engagement in other preventive or sexual health behaviors [15,17,18,19]. In the context of emerging infectious diseases and sexually associated infections, these interrelated dimensions may be highly relevant to understanding why some individuals seek vaccination immediately, whereas others delay or hesitate.

Importantly, vaccine hesitancy should not necessarily be treated as a single, uniform construct [20]. Classic definitions of vaccine hesitancy encompass both delay in acceptance and refusal of vaccination [21], and concerns among individuals without immediate willingness may overlap in some respects while differing in their relative prominence across respondents [13]. For example, hesitancy may reflect lower perceived susceptibility in some individuals, but greater emphasis on safety, access-related barriers, or more diffuse uncertainty in others. These differences need not represent wholly distinct mechanisms but may instead reflect variations in a broader preventive or risk-management orientation. In MSM, such orientations may also be expressed through other health-protective behaviors, including HIV or STI testing, PrEP or PEP use, and condom practices [22,23]. Accordingly, studying mpox vaccination willingness among MSM may benefit from attention not only to overall willingness, but also to variations within those lacking immediate willingness and its possible links to wider prevention orientations.

Research in this population also requires appropriate sampling strategies. Respondent-driven sampling (RDS) is a peer-referral method developed for hidden or hard-to-reach populations and has been widely used among MSM [24,25,26,27,28]. Compared with conventional convenience sampling, RDS may improve access to a wider range of participants through peer networks and reduce some forms of sampling bias when implemented appropriately [29,30,31]. In the present context, RDS is particularly relevant because MSM constitute a socially connected yet difficult-to-sample population in which health information, risk perception, and vaccine-related beliefs may be shaped within peer environments [32,33]. In addition to facilitating recruitment, the interpersonal structure generated through RDS can be incorporated into subsequent analyses to account for dependence arising from recruitment processes and to support a more robust assessment of associations between immediate willingness versus delayed/refused responses and individual behavioral and psychosocial factors in a city-level setting.

At the time of this study, no commercially available mpox vaccine was available on the Chinese mainland, and mpox prevention and control in China still relied primarily on non-vaccine measures, although vaccine development and clinical trials were ongoing [34]. In this context, evidence on mpox vaccination willingness among MSM is particularly relevant for informing future implementation strategies once vaccines become available. Existing studies have mainly focused on overall willingness or selected associated factors, with less attention to heterogeneity beyond a simple willing-versus-unwilling distinction and to the possibility that underlying concerns may differ in emphasis across individuals without immediate willingness to vaccinate. To address these gaps, this study used RDS to recruit MSM in Changsha, China, to assess mpox vaccination willingness; identify its sociodemographic, behavioral, and psychosocial correlates; and explore different concern profiles among those without immediate vaccination willingness. By examining both overall willingness and variation within the delayed/refused responses subgroup, this study seeks to inform more targeted vaccination communication and public health preparedness strategies for MSM and other hard-to-reach groups.

## 2. Materials and Methods

### 2.1. Respondent-Driven Sampling (RDS)

#### 2.1.1. RDS Procedure

This study was carried out by the Changsha Center for Disease Control and Prevention (CSCDC) between 1 September 2024 and 31 December 2024. Eligible participants were males aged 18 years or older who had engaged in anal or oral sex with another man in the past six months and had resided in Changsha for more than six months during the previous year.

RDS was employed to recruit participants. Details of the sampling procedure are available in the Supplementary Material Section S1. Five initial seeds were purposively selected by trained field investigators to reflect variation in key participant characteristics, including age, educational level, hukou status, and HIV status (one seed self-reported living with HIV). Each seed was provided with three recruitment coupons and instructed to invite up to three eligible peers from their MSM social networks. Recruited participants who completed the survey were subsequently given up to three coupons to continue peer recruitment. Each participant was permitted to complete the survey only once. All questionnaires were completed in person under one-to-one guidance from trained study staff after eligibility verification. When participants had difficulty understanding a question, clarification could be provided in real time. RDS recruitment and the survey implementation process are provided in the Supplementary Material Section S2.

Recruitment proceeded in successive waves until the target sample size was achieved. The recruitment chains are illustrated in Supplementary Material Section S3. Participants reported their personal network size (degree), defined as the number of MSM acquaintances (online and offline) whom they knew and considered eligible for study referral. The distribution of self-reported network degree was inspected prior to calculating RDS-II (Volz-Heckathorn) weights [35].

#### 2.1.2. RDS Diagnostics

Standard RDS diagnostics were performed to assess convergence, equilibrium, and potential recruitment dependence [30,36], with details provided in Supplementary Material Sections S4 and S5. RDS-II estimates across recruitment waves were examined graphically for four pre-specified variables: mpox vaccination willingness, age category, sexual orientation, and frequency of condomless anal intercourse in the past year. Recruiter-recruit mixing was also examined to assess potential homophily and chain-level dependence. One participant with a reported network size of zero was excluded from RDS weight computation but retained in unweighted analyses.

### 2.2. Variable Classification and Reprocessing

#### 2.2.1. Descriptive Variables

Descriptive variables were grouped into four domains:

1. Sociodemographic characteristics, including age category, education level, marital history, employment status, income category, and local hukou status.
2. Sexual behavior and health-related characteristics, including sexual orientation, condomless sex acts in the past year, erectile dysfunction (ED) drug use in the past year, ever used PrEP, ever diagnosed with any STI, and HIV diagnosis and testing history.
3. Mpox-related factors, including prior mpox infection, prior awareness of mpox, and mpox vaccination willingness.
4. Network-related measures, including total MSM acquaintances (online and offline), total online MSM acquaintances, and total sexual partners.

Categorical variables are presented as counts and percentages, while continuous variables with skewed distributions are summarized using medians and interquartile ranges (IQR). Detailed questionnaire wording, category definitions, and coding rules for all descriptive variables are provided in Supplementary Table S2. For the primary analysis, mpox vaccination willingness was categorized as immediate willingness versus delayed/refused responses. The latter combined “wait and see before deciding” and “refuse vaccination” because both indicated a lack of immediate willingness at the time of response. In addition, RDS-II weighted estimates were calculated for selected key population indicators to complement the unweighted descriptive results.

#### 2.2.2. Mpox Awareness and Psychosocial Constructs

Participants who reported prior awareness of mpox were subsequently administered additional five-point response-scale items across four conceptual domains: (1) information exposure frequency, (2) perceived transmission likelihood, (3) perceived risk-group susceptibility, and (4) vaccine-related beliefs. Information exposure frequency items were measured on a five-point frequency scale ranging from 1 (“never”) to 5 (“very often”). Items on perceived transmission likelihood and perceived risk-group susceptibility were measured on five-point perceived-likelihood scales ranging from 1 (“impossible”) to 5 (“very likely”), while vaccine-related belief items were measured on five-point agreement scales. Higher scores indicated greater frequency of information exposure, stronger perceived likelihood, or stronger agreement, depending on the item. These item sets were reduced into four psychosocial construct scores using principal component analysis (PCA) for subsequent analyses. Internal consistency was acceptable to good across the four item sets, with Cronbach’s alpha ranging from 0.725 to 0.859 and standardized alpha ranging from 0.726 to 0.860. Item wording, response scales, factor loadings, and additional PCA results are provided in Supplementary Table S3 and Supplementary Material Section S6.

#### 2.2.3. Delayed/Refused Responses Subgroup Analysis

To explore heterogeneity within the delayed/refused responses subgroup, analyses were restricted to participants who reported delayed/refused responses to receive mpox vaccination (n = 204). Concern profiles were derived from eight standardized items assessing vaccination-related concerns among participants with delayed/refused responses on five-point agreement scales. The unspecified open-ended “Other” response was excluded from clustering. Internal consistency of the eight concern items was acceptable, with a Cronbach’s alpha of 0.751 (standardized alpha: 0.752). Unsupervised clustering was performed using partitioning around medoids (PAM), and a three-cluster solution was selected based on internal validity and interpretability. Cluster labels were assigned post hoc according to dominant response patterns and were used for descriptive interpretation only. Original items and additional clustering details are provided in Supplementary Table S4 and Supplementary Material Section S7.

### 2.3. Modelling Strategy

We adopted a multi-step modeling strategy combining theory-driven covariate specification, penalized variable screening, and multiple regression frameworks to account for RDS design features and recruitment-chain dependence. Model outputs were summarized as odds ratios (ORs) with corresponding confidence intervals and visualized in forest plots for cross-model comparison. The overall analytic workflow is summarized in Supplementary Material Section S8.

#### 2.3.1. Outcome Definition

The primary outcome was mpox vaccination willingness, defined as a binary variable distinguishing immediate willingness to vaccinate from delayed/refused responses. This outcome definition was applied consistently across the main analytical framework, including penalized logistic regression for variable screening, multivariable logistic regression with recruitment chain-clustered robust standard errors, RDS-II weighted logistic regression, generalized estimating equations (GEE), and network-lag logistic regression. Accordingly, ORs from the main analyses represent the odds of immediate vaccination willingness compared with delayed/refused responses.

Subgroup analyses focused on participants with delayed/refused responses. The resulting concern-profile membership was used as the outcome variable in multinomial logistic regression models to examine sociodemographic and behavioral correlates of distinct concern patterns.

#### 2.3.2. Covariate Preparation

A conceptual framework summarizing the hypothesized relationships among mpox awareness, psychosocial dimensions, prevention-related behaviors, and vaccination willingness is available in Supplementary Material Section S6.

Variables of interest were harmonized prior to modeling, including recoding of categorical variables, construction of degree-related variables for RDS weighting, retention of recruitment-chain identifiers for clustered analyses, and log-transformation of network size measures. Additional technical details are provided in Supplementary Material Section S8.

#### 2.3.3. Psychosocial Constructs and Awareness-Conditioned Terms

Because psychosocial constructs were assessed only among participants who reported prior awareness of mpox, direct inclusion of PCA-derived scores would have introduced structural missingness for unaware individuals. To retain the full analytical sample while respecting this design feature, awareness-conditioned terms were constructed between mpox awareness status and each psychosocial component score (information exposure, transmission risk perception, risk group awareness, and vaccine beliefs). For participants without prior awareness, awareness-conditioned terms were set to zero by design, reflecting the conceptual assumption that psychosocial gradients operate only among individuals who are aware of mpox. Within the variable selection framework, these awareness-conditioned terms and prior mpox awareness, rather than the main psychosocial scores, were subjected to elastic net penalization. This specification allowed psychosocial gradients to contribute to the model conditionally on awareness status while avoiding redundancy or structural collinearity.

#### 2.3.4. Two-Stage Variable Selection

Variable selection followed a structured two-stage strategy combining forced-in covariates and penalized screening of extended predictors.

- Stage 1: Forced-in Core Covariates

A set of core variables was specified a priori and retained in all models regardless of penalized selection. These included age category, sexual orientation, log-transformed total MSM network size, condomless sex acts in the past year, and prior mpox awareness. These variables were selected based on theoretical relevance and the prior literature on vaccine hesitancy and mpox-related behaviors among MSM and were, therefore, not subjected to penalization [12,13,17,32,37].

- Stage 2: Penalized Screening of Extended Predictors

A secondary set of candidate variables was entered into elastic net penalized logistic regression (alpha = 0.5) for variable screening [38,39]. This penalized set included additional behavioral variables (PrEP use in the past year, ED drug use in the past year, and STI diagnosis history), additional networking measures (log-transformed online network size and sexual partner network size), and psychosocial gradient effects represented by four awareness-conditioned terms between mpox awareness and PCA-derived psychosocial scores.

#### 2.3.5. Multi-Model Framework (Main Analysis)

- Model 1: Logistic Regression with Chain-Cluster Robust Standard Errors

The primary association model was a multivariable logistic regression, accounting for recruitment-chain dependence induced by RDS, and inference was based on chain-clustered sandwich standard errors. Results were reported as ORs with 95% confidence intervals (CIs).

- Model 2A: RDS-II Weighted Logistic Regression

To account for unequal sampling probabilities inherent in RDS, we fitted an RDS-adjusted logistic regression model using RDS-II inverse-degree weights derived from reported personal network size. Log-transformed total network size was additionally retained as a predictor to assess its independent association with mpox vaccination willingness beyond its role in weighting. A sensitivity analysis excluding network size was conducted to evaluate potential redundancy between degree-based weighting and inclusion of network size as a covariate.

- Model 2B: Generalized Estimating Equations

To estimate population-averaged associations while explicitly modeling within-chain correlation, we fitted a generalized estimating equations (GEE) logistic model using an exchangeable working correlation structure and recruitment chain as the clustering unit. Robust standard errors were used for inference.

- Model 3: Network-Lag Logistic Regression

To evaluate potential peer influence along recruitment links, a recruiter-level mpox vaccination willingness variable was constructed by linking each participant to their recruiter’s immediate willingness versus delayed/refused responses. This network-lag term was added to the multivariable model. Inference was based on chain-cluster robust standard errors.

Cluster-robust logistic regression was treated as the primary inferential model, whereas the RDS-weighted, GEE, and network-lag models were used as complementary robustness analyses addressing different assumptions of the RDS-based data.

#### 2.3.6. Multinomial Regression of Concern-Profile Membership (Subgroup Analysis)

To examine correlates of distinct concern profiles within the delayed/refused responses subgroup, multinomial logistic regression was fitted with concern-profile membership as the outcome. Because this subgroup analysis was exploratory and based on a modest sample, covariates were selected pragmatically, considering substantive relevance, subgroup data sparsity, and consistency with the main analytical framework, including variables that were theoretically important or appeared informative in the primary regression models. The final model specification is described in Section 3. ORs with 95% CIs were derived from model coefficients. Sexual orientation and HIV-related behavioral status were recoded to reduce sparse cells, and complete-case analysis was applied within the delayed/refused responses subgroup (Supplementary Material Section S9 and Supplementary Table S7).

### 2.4. Statistical Analysis and Reporting

This cross-sectional study was reported in accordance with the Strengthening the Reporting of Observational Studies in Epidemiology (STROBE) guidelines [40]. Survey data were double-entered and validated using EpiData (version 3.1) to ensure data accuracy. All statistical analyses were conducted using R statistical software (version 4.4.0). Schematic diagrams were created using Mermaid (version 11.14.0). The final analytic dataset did not contain item-level missing data for the primary regression analyses. Sensitive responses such as “prefer not to say” were retained as substantive response categories rather than treated as missing values. Accordingly, the main analysis was based on the full analytic sample. For the RDS-weighted analysis (Model 2A), one record with a non-positive reported network degree was excluded because an RDS weight could not be computed.

## 3. Results

### 3.1. RDS Recruitment Diagnostics and Assessment of Sample Stability

A total of 405 MSM were recruited across five independent recruitment chains, with chain sizes moderately balanced. The largest chain contributed 113 participants (Chain 3), followed by Chain 5 (n = 94), Chain 1 (n = 84), Chain 4 (n = 61), and Chain 2 (n = 53), indicating no single chain dominated the sample. Recruitment progressed to substantial depths across all chains. The maximum recruitment wave reached 9 waves in Chain 5, 8 waves in Chain 3, and 6 to 7 waves in the remaining chains (Supplementary Material Section S1). Overall, the distribution of recruitment waves showed a clear concentration between waves 2 and 5, with diminishing contributions from later waves, suggesting stable recruitment dynamics rather than premature chain termination. The combination of multiple moderately sized chains and sufficient recruitment depth supports the adequacy of the RDS process to reach beyond initial seeds and reduce seed-dependent bias.

Visual inspection of RDS-II convergence plots (Supplementary Material Section S4) indicated that mpox vaccination willingness, age category, sexual orientation, and condomless sex frequency stabilized after early recruitment waves, suggesting that equilibrium was achieved and that estimates were not driven by initial seeds. Recruiter-recruit similarity analyses (Supplementary Table S1 and Supplementary Material Section S5) provided limited evidence of statistically significant assortative recruitment across key variables. In addition, random-intercept models showed negligible between-chain variance in mpox vaccination willingness (ICC ≈ 0), suggesting little clustering at the recruitment-chain level. Collectively, these findings suggest that mpox vaccination willingness was not materially structured by recruitment dynamics, supporting the internal validity of subsequent regression analyses.

### 3.2. Participant Characteristics and Mpox Vaccination Willingness

A total of 405 MSM participants aged 18–61 years were recruited (median age: 26 years), without self-reported mpox infection history. As shown in Table 1 (Section A), the majority were aged 25–34 years (53.09%), had at least a bachelor’s degree (46.67%), were unmarried (93.83%), and over half were employed (56.30%). Approximately two-thirds did not hold local hukou.

Sexual orientation was predominantly self-reported as homosexual (82.22%). Half of the participants reported no condomless sex in the past year, while a minority reported frequent (≥6 times) condomless sex. PrEP use and erectile dysfunction drug use were reported by fewer than one-fifth of participants. Five participants self-reported living with HIV, and most participants were HIV-negative and had tested within the past three months (64.20%).

Prior awareness of mpox was high (92.59%), and mpox vaccination willingness was nearly evenly distributed (201 immediate vs. 204 delayed/refused).

Network characteristics are presented in Table 1 (Section B), with participants reporting a median of 15 MSM acquaintances and a median of one sexual partner.

RDS-II weighted estimates for key population indicators showed a similar distribution: 48.6% were aged 25–34 years, 41.6% were aged 18–24 years, and 9.8% were aged ≥35 years. Weighted proportions indicated that 77.5% identified as homosexual, 88.7% reported prior mpox awareness, and 47.3% reported immediate vaccination willingness.

### 3.3. Concern Profiles Within the Delayed/Refused Responses Subgroup

As shown in Supplementary Table S4, among participants with delayed/refused responses (n = 204), perceived low infection risk and safety concerns were the most prominent reasons. A total of 73.53% agreed or strongly agreed that they were unlikely to be infected, and 73.04% expressed concern about vaccine side effects. Structural barriers were also frequently endorsed, including concerns about procedural complexity or time burden (58.82%) and high vaccination cost (58.33%). Privacy-related concerns were present but less prominent, with 42.65% expressing concern about MSM identity disclosure and 29.90% about HIV/STI testing-related privacy. Perceived limited vaccine effectiveness was endorsed by 32.35% of participants in the delayed/refused responses subgroup, while 26.47% reported concerns about medical contraindications.

This distribution of concerns suggests that delayed/refused responses were shaped primarily by low perceived susceptibility, safety concerns, and practical barriers, while also showing variation in the relative prominence of specific concerns. On this basis, clustering analysis was conducted to explore internal variation within the delayed/refused responses subgroup.

**Figure 1:**
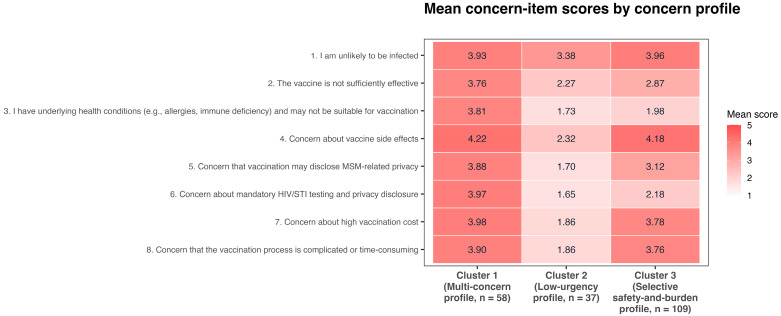
Heatmap of mean concern-item scores across the concern profiles within the delayed/refused responses subgroup (n = 204). Values for clusters 1–3 represent mean scores on five-point agreement scales (range: 1–5), with higher values indicating stronger endorsement of the stated concern. Cluster labels were assigned post hoc for descriptive interpretation only.

Unsupervised clustering identified three concern profiles among participants with delayed/refused responses. Perceived low infection risk was highly endorsed across all three profiles, suggesting a shared concern structure within the delayed/refused response subgroup. The profiles were differentiated less by the presence of this concern than by the relative prominence of additional barriers. The distinguishing features of the three retained concern profiles are shown visually in Figure 1 and are summarized below.

Cluster 1 (Multi-concern profile, n = 58) showed consistently high levels of concern across nearly all assessed dimensions (mean scores 3.76–4.22). In addition to high endorsement of perceived low infection risk (mean = 3.93), this cluster also showed elevated concerns regarding vaccine effectiveness, contraindications, side effects, privacy, financial cost, and procedural burden. This profile suggests delayed vaccination characterized by broadly elevated barriers across domains rather than by any single dominant concern.

Cluster 2 (Low-urgency profile, n = 37) showed the lowest overall level of concern across most dimensions (mean scores 1.65–3.38). Perceived low infection risk remained the highest-rated item in this cluster (mean = 3.38), whereas safety, structural, privacy-related, effectiveness, and contraindication concerns were comparatively weak. This profile suggests delayed vaccination is characterized less by multiple elevated barriers than by limited urgency or passive deferral.

Cluster 3 (Selective safety-and-burden profile, n = 109) showed high endorsement of vaccine side effects (mean = 4.18), financial cost (3.78), procedural burden (3.76), and perceived low infection risk (3.96), while concerns about vaccine effectiveness, contraindications, and HIV/STI testing-related privacy were comparatively lower. Unlike Cluster 1, this profile was not characterized by uniformly elevated concern across all domains, but by a more selective pattern centered on safety and practical barriers in the context of a shared low-risk perception.

### 3.4. Two-Stage Variable Selection Results

Elastic net penalized logistic regression identified seven additional predictors beyond the pre-specified core covariates. Retained variables included behavioral factors (ever use of PrEP, ED drug use in the past year, and history of STI diagnosis) and all four awareness-conditioned psychosocial terms between prior mpox awareness and PCA-derived component scores (information exposure frequency, perceived transmission likelihood, perceived risk-group susceptibility, and vaccine-related beliefs). The final multivariable model, therefore, comprised the five forced-in core variables (age category, sexual orientation, log-transformed total network size, condomless sex acts in the past year, and prior mpox awareness) together with the seven penalization-selected predictors.

### 3.5. Primary Regression Model Results

**Figure 2:**
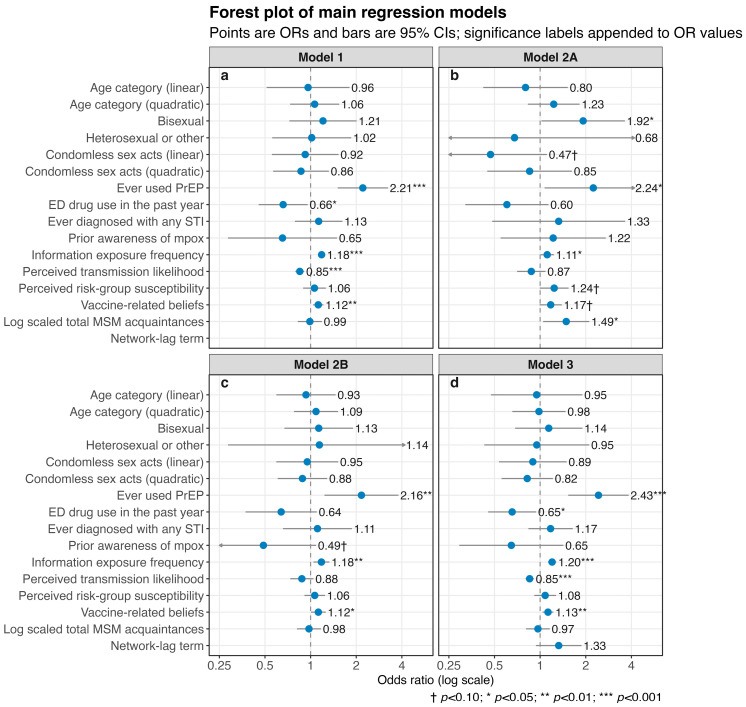
Forest plot of primary regression models for immediate mpox vaccination willingness. (**a**) Associations estimated using cluster robust logistic regression (Model 1); (**b**) associations estimated using RDS-II weighted logistic regression (Model 2A); (**c**) associations estimated using generalized estimating equations (Model 2B); (**d**) associations estimated using network lag logistic regression (Model 3). Blue points indicate odds ratios, and horizontal lines indicate 95% confidence intervals. Arrows indicate that the corresponding confidence intervals extend beyond the plotted range. For clarity, abbreviated variable labels are displayed in the figure. Full model terms correspond to the following: age category (linear and quadratic polynomial terms); sexual orientation (bisexual vs. homosexual; heterosexual or other vs. homosexual [reference]); condomless sex acts in the past year (linear and quadratic terms); ever used PrEP (yes vs. no); ED drug use in the past year (yes vs. no); ever diagnosed with any STI (yes vs. no); prior awareness of mpox (yes vs. no [reference]); four awareness-conditioned terms between prior mpox awareness and psychosocial gradients (information exposure frequency PC1, perceived transmission likelihood PC1, perceived risk-group susceptibility PC1, vaccine-related beliefs PC1); log-transformed total MSM acquaintances (online and offline); and the recruiter-level network-lag term.

Across the primary models shown in Figure 2, including cluster robust logistic regression (Model 1), RDS-II weighted logistic regression (Model 2A), generalized estimating equations (Model 2B), and the network lag model (Model 3), the overall pattern of associations was broadly similar. Behavioral and psychosocial variables showed largely directionally consistent associations across alternative model specifications, although statistical significance varied under different weighting assumptions.

Ever use of PrEP was the most stable correlate of immediate vaccination willingness, with approximately twofold higher odds across the primary models (Model 1, OR = 2.21, 95% CI: 1.51 to 3.24; Model 2A, OR = 2.24, 95% CI: 1.07 to 4.70; Model 2B, OR = 2.17, 95% CI: 1.24 to 3.80; Model 3, OR = 2.43, 95% CI: 1.53 to 3.84). Although this association attenuated slightly and became marginal in the RDS weighted sensitivity model (Supplementary Table S4), its magnitude and direction remained consistent.

Awareness-conditioned psychosocial terms conditional on prior mpox awareness also showed informative associations with vaccination willingness. Higher information exposure frequency (PC1 × awareness) was positively associated with immediate vaccination willingness in Models 1, 2A, 2B, and 3, although the association was attenuated and no longer statistically significant in the RDS weighted sensitivity model. Similarly, the awareness-conditioned term for vaccine-related beliefs was positively associated with immediate willingness in Models 1, 2B, and 3, but only marginally in the weighted models. By contrast, perceived transmission likelihood was inversely associated with immediate willingness in Models 1 and 3 and showed borderline significance in the RDS weighted sensitivity model. Perceived risk group susceptibility showed weaker and less consistent evidence of association across models.

In the primary RDS-II weighted model, Model 2A, log-transformed total network size was positively associated with immediate vaccination willingness (OR = 1.49, 95% CI: 1.05 to 2.11). Because this variable was also closely related to the inverse degree weighting used in RDS-II estimation, a sensitivity analysis excluding network size was conducted. In that model, the principal behavioral and psychosocial associations remained directionally consistent, with effect estimates of broadly similar magnitude, although statistical precision was reduced for some variables. This pattern suggests that the principal findings were broadly robust to alternative weighting specifications.

The recruiter linked network lag term was not statistically significant in Model 3. Basic sociodemographic variables showed little evidence of independent association with vaccination willingness across the primary models.

### 3.6. Multinomial Regression Results for Concern Profiles

**Figure 3:**
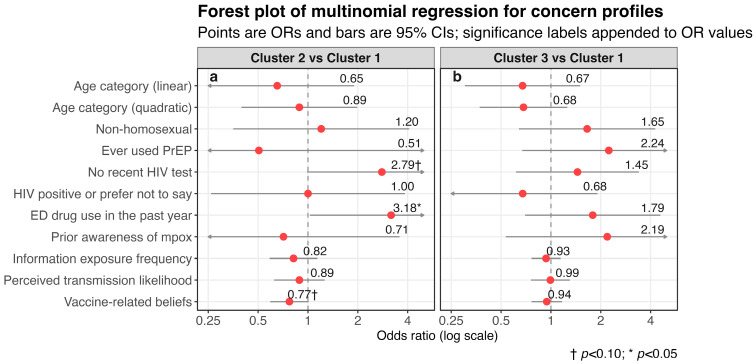
Forest plot of multinomial regression results for concern profiles. (**a**) Cluster 2 compared with Cluster 1 as the reference group; (**b**) Cluster 3 compared with Cluster 1 as the reference group. Points indicate odds ratios, and horizontal lines indicate 95% confidence intervals. Red points indicate odds ratios, and horizontal lines indicate 95% confidence intervals. Arrows indicate that the corresponding confidence intervals extend beyond the plotted range. For clarity, abbreviated variable labels are displayed in the figure. Full model terms correspond to the following: age category (linear and quadratic polynomial terms); sexual orientation (non-homosexual vs. homosexual [reference], combining bisexual and heterosexual or other); ever used PrEP (yes vs. no); HIV-related behavioral status (no recent test vs. recent test [reference]; positive or prefer not to say vs. recent test); ED drug use in the past year (yes vs. no); prior awareness of mpox (yes vs. no [reference]); and selected awareness-conditioned terms between prior mpox awareness and psychosocial gradients (information exposure frequency PC1, perceived transmission likelihood PC1, vaccine-related beliefs PC1).

As an exploratory extension of the primary analyses, the multinomial regression model included age category and sexual orientation as core sociodemographic variables; ever use of PrEP, ED drug use in the past year, and HIV-related behavioral status as indicators of prevention engagement and sexual-health context; and prior mpox awareness together with selected PCA-derived awareness-conditioned psychosocial terms for information exposure frequency, perceived transmission likelihood, and vaccine-related beliefs. Multinomial regression analyses yielded limited evidence of variables independently associated with concern-profile membership (Figure 3; Supplementary Table S6). For Cluster 2 relative to Cluster 1, ED drug use in the past year was positively associated with Cluster 2 membership (OR = 3.18, 95% CI: 1.03 to 9.82, *p* = 0.045). Two additional borderline associations, defined as *p* < 0.10, were observed for Cluster 2. Compared with participants who had undergone recent HIV testing, those reporting no recent test showed higher odds of belonging to Cluster 2 (OR = 2.80, 95% CI: 0.99 to 7.91, *p* = 0.053). The awareness-conditioned term of vaccine-related beliefs, PC1, showed a marginal inverse association with Cluster 2 membership (OR = 0.77, 95% CI: 0.59 to 1.01, *p* = 0.058). No statistically significant or borderline associations were observed for Cluster 3 relative to Cluster 1.

## 4. Discussion

In this RDS-recruited sample of MSM in Changsha, willingness to receive mpox vaccination was almost evenly divided between immediate and delayed/refused responses, indicating that lack of immediate willingness was not a marginal phenomenon in this population. Across alternative modeling strategies, ever use of PrEP showed the most consistent association with immediate vaccination willingness, whereas several psychosocial gradients conditional on prior mpox awareness were directionally similar but less stable across models. By contrast, chain-level clustering and recruiter-linked network effects appeared limited, suggesting that observed variation in vaccination willingness was more likely related to individual-level behavioral and psychosocial factors than to specific recruitment-chain measures examined here. Among participants without immediate willingness, hesitancy-related concerns did not follow a single uniform pattern. The three identified clusters shared low perceived infection risk as a common concern but differed in the relative prominence of additional barriers, including broadly elevated cross-domain profile, low-urgency profile, and more selective safety- and burden-related profile. Exploratory multinomial analyses further suggested that, relative to the multi-concern profile, the low-urgency profile may be related to a weaker alignment between ongoing sexual activity and preventive engagement.

Our previous survey, conducted when the mpox outbreak first emerged locally in 2023, suggested very high vaccination acceptance among males living with HIV, including both previously and newly diagnosed cases, exceeding 90%, whereas acceptance was lower, though still above 70%, among HIV-suspected males who had not met confirmatory criteria [12]. By contrast, in the present RDS-based study targeting MSM, only about half of the participants reported immediate willingness to vaccinate, and very few participants self-reported living with HIV. This contrast is compatible with the possibility that HIV status may be related to mpox vaccination willingness, but direct comparison between the two surveys should be cautious because they differed in sampling strategy, target population, survey timing, and HIV ascertainment. In particular, the 2023 survey included laboratory-based HIV classification, whereas HIV status in the present study was self-reported. Even so, the overall pattern remains broadly consistent with our previous findings [12], and with evidence from large MSM surveys and systematic reviews reporting similar associations [17,41,42,43].

Another possible explanation for the lower willingness observed in the present study, compared with the earlier survey, is survey timing. A systematic review of studies published between January 2022 and November 2023 reported that willingness to receive mpox vaccination among MSM ranged from approximately 50% to over 80% across countries, with pooled estimates around 60% during the early outbreak period [17]. In China, several surveys conducted during the early outbreak phase reported relatively high acceptance, often ranging from about 70% to over 90% [11,44,45,46,47,48]. More recent data remain limited. It is plausible that, as the outbreak became less prominent and mpox received less sustained public attention, the perceived urgency of vaccination also declined. This interpretation is supported by recent meta-analytic evidence suggesting that mpox vaccine acceptance declined over time, with pooled estimates decreasing from approximately 85% in studies published in 2022 to around 62% in 2024, possibly reflecting reduced perceived risk as the outbreak subsided [15]. However, such comparisons should still be interpreted cautiously because vaccine availability, rollout context, and healthcare access differed across settings. Against this background, individual-level informational and psychosocial factors may become particularly relevant. Prior research on health communication and vaccination behavior suggests that exposure to health information can improve vaccination intentions and support preventive decision-making [13,49,50]. In our previous 2023 survey, the psychosocial factor with high loadings for mpox risk-group awareness was positively associated with higher vaccination acceptance among males living with or suspected of HIV infection [12]. In the current survey, by contrast, greater mpox-related information exposure frequency among MSM who were already aware of mpox showed a more consistent association with immediate willingness across all primary models, while stronger vaccine-related beliefs among mpox-aware MSM were also associated with immediate responses in several models. These findings suggest that, even during periods of lower epidemic salience, continued engagement with disease-related information may remain relevant to vaccination willingness [51,52] and that the psychosocial dimensions most closely related to vaccination willingness may shift across epidemic contexts [53,54]. On top of this, the inverse association observed for the awareness-conditioned perceived transmission likelihood term should be interpreted with caution. This construct associates a stronger endorsement that mpox may be transmitted through multiple routes. In practice, higher scores may capture a broader or less differentiated perception of transmission possibilities, or a more diffuse sense of uncertainty about how transmission occurs, rather than a more focused sense of immediate personal urgency. This interpretation is consistent with reports that knowledge gaps and ambiguity regarding mpox transmission remain common [55].

In addition to the psychosocial constructs measured conditional on prior mpox awareness, HIV-related behaviors also appeared relevant to vaccination willingness among MSM in Changsha. Our primary modelling results consistently identified ever use of PrEP as a correlate of vaccination willingness, which is broadly consistent with prior studies showing higher mpox vaccine willingness and uptake among PrEP users [17,42,56]. Rather than simply representing stronger general health consciousness, this association may help inform a more specific prevention orientation. As a biomedical strategy aimed at preventing HIV acquisition, PrEP use could be associated with greater acceptance of biomedical prevention, higher perceived susceptibility within sexual-health networks, and closer engagement with sexual-health services where vaccination information and access are available [42,43,57,58,59,60]. Previous evidence has also linked mpox vaccination willingness to other markers of sexual-health risk or engagement, including a higher number of sexual partners and prior STI diagnosis, although these associations have not always been consistent across studies [15,17,19,43,56]. The absence of similarly robust associations for these other markers in our study does not necessarily contradict this interpretation, as they may capture sexual-health risk or prevention orientation less specifically than PrEP use. Condom use has also been examined in some studies, but the evidence appears less consistent, and it was likewise not significantly associated with vaccination willingness in our study. One possible explanation is that condoms provide only partial protection against mpox, which can also be transmitted through close skin-to-skin and mouth-to-skin contact outside the areas covered by condoms [61].

Prior vaccine hesitancy research has applied person-centered methods, including latent class analysis and cluster analysis, to identify subgroups or concern profiles rather than treating hesitancy as a single homogeneous construct. Examples include latent class analyses of HPV vaccine worries, COVID-19 vaccine hesitancy, and parental vaccine hesitancy more broadly [62,63,64]. However, the use of cluster-analytic approaches in mpox vaccination research remains limited. In our study, among respondents with delayed/refused responses, perceived low infection risk was highly endorsed across all three profiles, suggesting a common concern across those lacking immediate willingness. This pattern is largely consistent with classic vaccine hesitancy frameworks in which complacency reflects low perceived disease risk and low perceived need for vaccination [54]. The between-profile differences appeared to lie less in the presence of this common core than in the relative prominence of other concern dimensions. One profile was characterized by elevated concern across domains, another by generally lower levels of concern beyond low perceived risk, and a third by a more selective emphasis on safety- and burden-related concerns. The low-urgency profile may, therefore, be better understood as supporting reduced urgency or passive deferral rather than predominantly fear-based concern. This interpretation is tentatively supported by the multinomial regression, in which lower vaccine-related beliefs and no HIV testing in the past three months showed marginal associations with this profile, while ED drug use in the past year was positively associated with Cluster 2 membership and points to ongoing sexual activity. Relative to the multi-concern profile, the low-urgency profile may be compatible with a weaker alignment between ongoing sexual activity and preventive engagement.

From a public health perspective, these findings might have implications beyond explaining willingness patterns alone. First, the consistent role of mpox-related information exposure is suggestive of the potential value of targeted digital communication that is timely, behaviorally relevant, and tailored to MSM communities [65]. Second, the observed patterns of mpox vaccination willingness could be relevant to broader patterns of sexual-health engagement [66]. Third, the heterogeneity within the delayed/refused group may help inform subgroup-sensitive, community- and peer-informed approaches that address different combinations of low urgency, safety concerns, privacy-related concerns, and practical barriers. Considered together, these findings support communication and prevention strategies that are not only risk-informed but also subgroup-sensitive and embedded within broader sexual-health service frameworks, in which HIV, STIs, and mpox are approached together.

This study has several notable strengths. First, the use of RDS, together with standard RDS diagnostics and additional assessments of recruitment dependence, was intended to improve community-based access to MSM in Changsha beyond what might be captured through facility-based or venue-based convenience sampling while also allowing evaluation of whether recruitment-chain structure materially influenced the primary outcome. Second, the questionnaire assessed key vaccine-relevant dimensions on graded five-point scales, which avoided unnecessary dichotomization and enabled a more nuanced analysis of variation within the study population. Third, the primary outcome distinguished immediate willingness from delayed/refused responses, rather than reducing uptake intention to a simple yes-or-no contrast. This specification was better aligned with the conceptual scope of vaccine hesitancy, as defined by WHO SAGE to include both delayed acceptance and refusal [21], and it also enabled further examination of heterogeneity within the subgroup lacking immediate vaccination willingness through profile-based analysis. Finally, the adoption of multiple modelling strategies increased confidence in the robustness of the primary associations.

Several limitations should also be acknowledged. First, the cross-sectional design precludes causal inference, and the observed associations should not be interpreted as evidence of temporal or causal relationships. Second, the study was conducted in a single city, which may limit the transferability of the findings to MSM populations in other contexts that differ in healthcare accessibility, community connectedness, and local sexual-health service provision, given that regional variation in vaccination-related attitudes and psychosocial barriers across mainland China has not been well characterized. This limitation could be especially relevant for rural MSM populations. Third, although standard RDS diagnostics and our additional assessments suggested broadly stable recruitment patterns for the key measured variables, these checks cannot verify all RDS assumptions, and residual bias due to self-reported network size, seed dependence, or unmeasured homophily cannot be excluded. RDS may also underrepresent MSM who are socially isolated, weakly connected to recruitment chains, or otherwise outside the active peer networks through which recruitment occurred [67]. In the absence of a conventional sampling frame, such subgroups may be difficult to capture through alternative recruitment strategies as well, and may require additional community-based outreach, targeted field investigation, or dedicated studies to be adequately captured. Fourth, key measures, including sexual behavior, HIV-related variables, mpox awareness, and vaccination willingness, were self-reported and may, therefore, be subject to reporting bias. For some variables, this could include recall error. For HIV status, however, the more relevant concern is likely underreporting or nondisclosure in a sensitive social context rather than simple memory failure because laboratory confirmation was not part of study enrollment. Only five participants reported living with HIV, limiting the precision and statistical power available to evaluate self-reported HIV-positive status as an independent correlate of vaccination willingness [68,69]. Fifth, the multinomial analysis within the delayed/refused response subgroup was based on a modest sample size. Although this subgroup comprised roughly 200 participants, the effective information available for estimation was more limited after division into multiple outcome categories and inclusion of predictors with several categorical levels. To reduce instability and the risk of quasi-complete separation, selected sparse categories were recoded prior to modeling. However, some subgroup-specific estimates remained imprecise, with relatively wide confidence intervals. Accordingly, this subgroup analysis should be interpreted as exploratory and hypothesis-generating rather than definitive. Finally, stated vaccination willingness does not necessarily translate into actual vaccine uptake [43], particularly when access, cost, convenience, or changing risk perception affect real-world behavior.

## 5. Conclusions

This study suggests that analyses of mpox vaccination willingness among MSM in Changsha could benefit from considering not only overall willingness, but also variation within the delayed/refused responses subgroup. The near-equal distribution between immediate willingness and delayed/refused responses indicates that lack of immediate willingness was not a marginal phenomenon in this population. Vaccination willingness appeared to be related more to prevention-related behaviors and psychosocial factors than to basic sociodemographic profile alone. Within the delayed/refused responses subgroup, perceived low infection risk was a common concern, whereas the relative prominence of other barriers varied across profiles. These findings may help inform mpox vaccination preparedness by highlighting the potential value of risk communication, subgroup-sensitive approaches to vaccine-related barriers, and integrated prevention efforts in which HIV, STIs, and mpox are addressed within a shared sexual-health framework.

## Figures and Tables

**Table 1:** Participant characteristics and mpox vaccination willingness (n = 405).

**Section A: Categorical Variables**		
**Variables**	**Number**	**Percentage (%)**
**Age category**		
18–24	143	35.31
25–34	215	53.09
≥35	47	11.60
**Education levels**		
Senior high or below	41	10.12
Professional training college	132	32.59
Bachelor’s degree	189	46.67
Master’s degree or above	43	10.62
**Married or once married**		
Yes	25	6.17
No	380	93.83
**Employment status**		
Yes	228	56.30
No	177	43.70
**Income category (RMB)**		
<2000	71	17.53
2000–4999	91	22.47
5000–7999	149	36.79
≥8000	90	22.22
Prefer not to say	4	0.99
**Local hukou**		
Yes	135	33.33
No	270	66.67
**Sexual orientation**		
bisexual	63	15.56
homosexual	333	82.22
heterosexual or other	9	2.22
**Condomless sex acts (past year)**		
Never	206	50.86
1 to 5 times	155	38.27
6 times and over	44	10.86
**ED drug use (past year)**		
Yes	70	17.28
No	335	82.72
**Ever used PrEP**		
Yes	60	14.81
No	345	85.19
**Ever diagnosed with any STI**		
Yes	78	19.26
No	327	80.74
**HIV diagnosis and testing history**		
HIV negative but not tested in 3 months	104	25.68
HIV negative and tested in 3 months	260	64.20
HIV positive	5	1.23
Prefer not to say	36	8.89
**Prior awareness of mpox**		
Yes	375	92.59
No	30	7.41
**Mpox vaccination willingness**		
delayed/refused	204	50.37
immediate	201	49.63
**Section B: Continuous Variables**		
**Variables**	**Median**	**IQR**
Total MSM acquaintances (online and offline)	15	(7, 43)
Total online MSM acquaintances	5	(2, 18)
Total sexual partners	1	(1, 4)

## Data Availability

The data presented in this study are not publicly available due to privacy and ethical restrictions related to the sensitive nature of the survey data. De-identified data may be made available by the corresponding authors upon reasonable request and subject to approval by the relevant ethics committee and applicable data access requirements.

## Supplementary Materials

The following supporting information can be downloaded at: https://www.mdpi.com/article/10.3390/vaccines14050428/s1, Supplementary Table S1: Recruiter–Recruit Mixing Patterns for Key Variables (N = 405), Supplementary Table S2: Original Survey Questions and Coding Rules for Categorical and Continuous Variables, Supplementary Table S3: Individual Item Wording and Response Scales of Four Mpox Awareness Domains (N = 375), Supplementary Table S4: Concerns for Delaying or Refusing Vaccination (N = 204), Supplementary Table S5: Mean Concern-item Scores across Candidate PAM Solutions and Ward Hierarchical Clustering in the Delayed/Refused Responses Subgroup, Supplementary Table S6: Multivariable Associations with Acceptability of Mpox Vaccination, Supplementary Table S7: The Crosstables of Collapsed Variables in Multinomial Logistic Regression (N = 204), Supplementary Table S8: Multinomial Logistic Regression of Vaccine Hesitancy Cluster Membership Within the Concern Profiles (Reference = Cluster 1; N = 58); Supplementary Material Section S1: RDS Sample Size Estimation, Supplementary Material Section S2: RDS Recruitment and Survey Implementation Process, Supplementary Material Section S3: RDS Recruitment Chain Structure and Wave Depth, Supplementary Material Section S4: RDS Equilibrium and Degree Diagnostics, Supplementary Material Section S5: RDS Homophily Diagnostics, Supplementary Material Section S6: Mpox Awareness and Psychosocial Constructs, Supplementary Material Section S7: Concern Profiles within the Delayed/Refused Responses Subgroup, Supplementary Material Section S8: Modelling Strategy Overview and Supplementary Technical Details, Supplementary Material Section S9: Recoded Variables in Multinomial Regression for Concern-Profile Membership; Supplementary Figure S1. RDS participant flow and survey implementation process, Supplementary Figure S2. RDS Recruitment Chains of Five Seeds (N = 405), Supplementary Figure S3. The Distribution of Log-transformed Self-reported Network Size (RDS Degree), Supplementary Figure S4. Convergence Plots of Four Key Study Variables, Supplementary Figure S5. Conceptual framework linking mpox awareness, psychosocial dimensions, prevention-related behaviors, and vaccination willingness, Supplementary Figure S6. Average Silhouette Width across Candidate PAM Solutions (k = 2–4) for the Delayed/Refused Responses Subgroup, Supplementary Figure S7. Schematic overview of the analytic strategy. Refs. [30,31,36,70,71,72,73] are cited in Supplementary Materials.

## Abbreviations

The following abbreviations are used in this manuscript:

ACAM2000	second-generation smallpox vaccine
AIDS	acquired immunodeficiency syndrome
CI	confidence interval
CSCDC	Changsha Center for Disease Control and Prevention
ED	erectile dysfunction
GEE	generalized estimating equations
HIV	human immunodeficiency virus
ICC	intraclass correlation coefficient
IQR	interquartile range
JYNNEOS	third-generation smallpox and mpox vaccine
MSM	men who have sex with men
MPXV	monkeypox virus
NGO	non-governmental organization
OR	odds ratio
PAM	partitioning around medoids
PCA	principal component analysis
PC1	first principal component
PEP	post-exposure prophylaxis
PrEP	pre-exposure prophylaxis
RDS	respondent-driven sampling
RDS-II	Volz-Heckathorn respondent-driven sampling estimator
STI	sexually transmitted infection
STROBE	Strengthening the Reporting of Observational Studies in Epidemiology
USD	United States dollar
WHO	World Health Organization
