# Supplementary material for: Mpox Vaccination Willingness, Concern Profiles, and Associated Factors Among Men Who Have Sex with Men in Changsha, China
Source: Vaccines (Basel). 2026 May 10;14(5):428. doi: 10.3390/vaccines14050428 (PMC13211385; doi:10.3390/vaccines14050428)
Supplement: Supplementary file 1 [file vaccines-14-00428-s001.zip › Supplementary Materials.pdf]

Supplementary Materials

S1. RDS Sample Size Estimation

Because MSM constitute a hard-to-reach population without a conventional sampling frame, sample size planning could not rely on assumptions of independent simple random sampling alone. Accordingly, the conventional single-proportion formula was used only to obtain a base sample size for prevalence estimation, which was then adjusted for the design effect introduced by network-based recruitment under RDS [1]. Details of the calculation are provided below for transparency.

Using the standard formula:

$$N = \frac{Z_{1-\alpha/2}^2 \times p(1-p)}{d^2} \times \mathrm{DEFF}$$

Assuming a conservative prevalence of 50% (p = 0.50), a 95% confidence level ($Z_{1-\alpha/2} = 1.96$), and a margin of error of 7% (d = 0.07) as a pragmatic precision target, the base sample size was approximately 196. This was then multiplied by a design effect of 2.0 (DEFF = 2) to account for network-based dependence under RDS, yielding a target sample size of approximately 392 participants. A total of 405 eligible participants were ultimately verified, enrolled, and completed the survey.

All questionnaires were completed in person under one-to-one guidance from trained study staff after eligibility verification. When participants had difficulty understanding a question, clarification could be provided in real time. One participant with a reported network size of 0 was excluded from RDS-weighted analyses because positive network size is required for weighting but was retained in descriptive and all non-RDS-weighted analyses.

S2. RDS Recruitment and Survey Implementation Process

In this study, five seeds with different backgrounds were selected to initiate recruitment. Seeds were chosen to enhance diversity of network entry points and were expected to have relatively high levels of activity within MSM social networks in Changsha, good communication ability, and willingness to cooperate with study procedures. To reduce overlap between initial recruitment chains, seeds were not selected from individuals who already knew one another.

Supplementary Figure S1 illustrates the standardized on-site implementation process for the RDS survey. Participants arrived with recruitment coupons, underwent coupon validity verification and eligibility screening, and provided written informed consent before survey administration. Eligible participants then completed the core questionnaire interview in person under one-to-one guidance from trained study staff, together with personal network size assessment. Voluntary biological testing (e.g., HIV/syphilis) was offered after completion of the interview. A primary participation

incentive of 50 RMB was then provided, consistent with common recruitment practice in RDS studies. In addition, participants could receive a secondary recruitment incentive of 10 RMB for each successfully referred peer who met eligibility criteria and completed the survey, for up to three peers. Thus, the maximum total compensation available to a participant was 80 RMB. Although modest incentives may influence participation decisions, in this study they were implemented as part of the standardized sampling and recruitment procedure rather than as study exposures of analytic interest [2-4].

Recruitment coupons were subsequently generated and explained for peer recruitment. As part of the standardized RDS procedure, coupons served as the sole tracing mechanism for recruitment links. Each coupon carried a unique identifier incorporating recruiter identity and issuance batch, allowing staff to link the coupon presented by an incoming participant with the new coupons subsequently issued for onward recruitment. Unique numbering also functioned as an anti-fraud measure. Coupons were assigned an explicit expiry period (e.g., approximately four weeks) to facilitate wave progression and reduce delays in recruitment. Each eligible participant was typically provided with up to three recruitment coupons and was permitted to complete the survey only once.

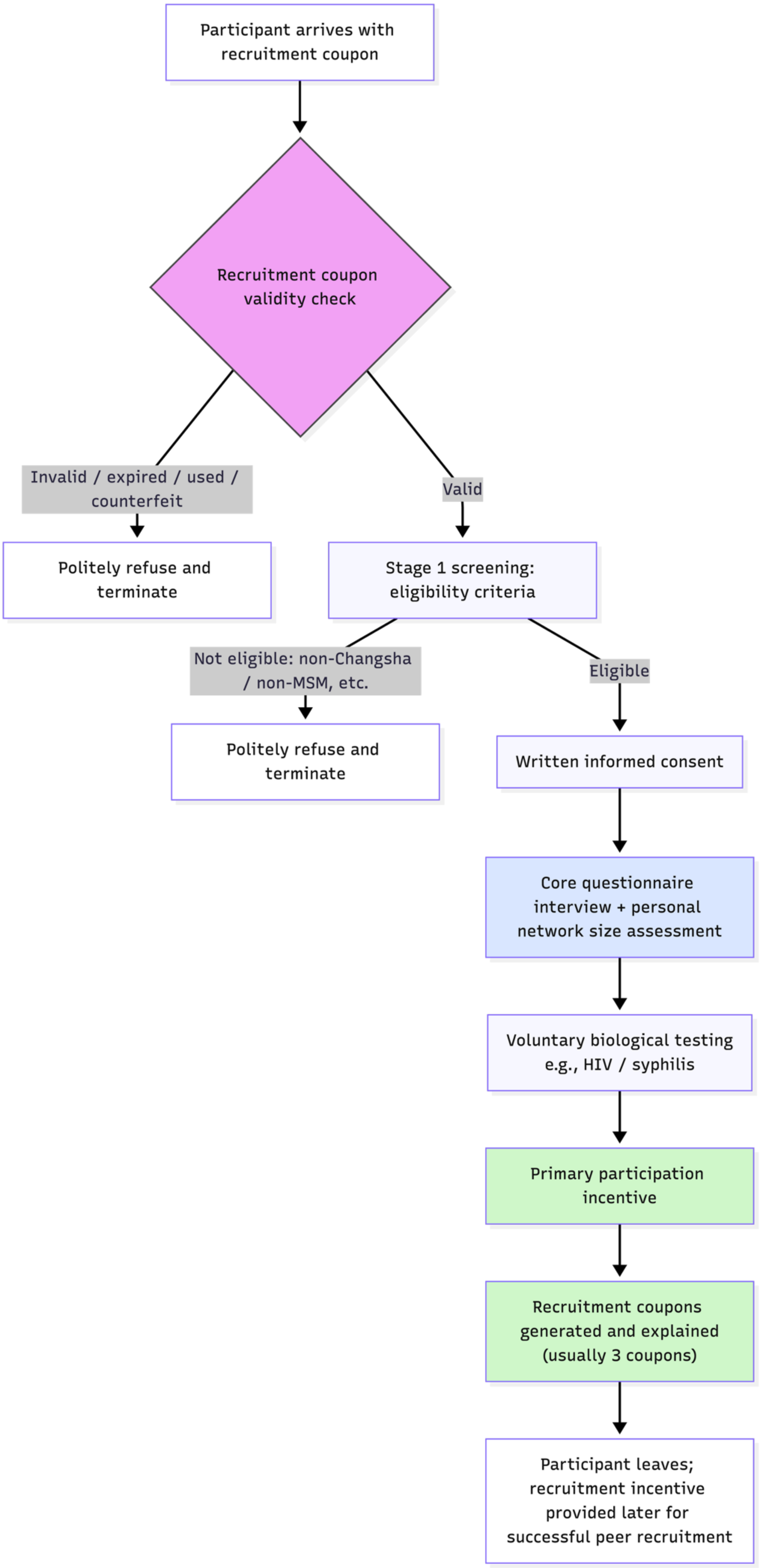

Supplementary Figure S1. RDS participant flow and survey implementation process

S3. RDS Recruitment Chain Structure and Wave Depth

Supplementary Figure S2 displays five independent RDS recruitment chains comprising 405 participants in total. Chain sizes are moderately balanced, ranging from 53 (Chain 2) to 113 (Chain 3), with no single chain dominating the sample. Recruitment depth was adequate across chains, reaching maximum waves of 6-9, with Chain 5 extending to the deepest level (9 waves). Overall, the trees show sustained multi-wave propagation from initial seeds, supporting sufficient recruitment penetration beyond early waves.

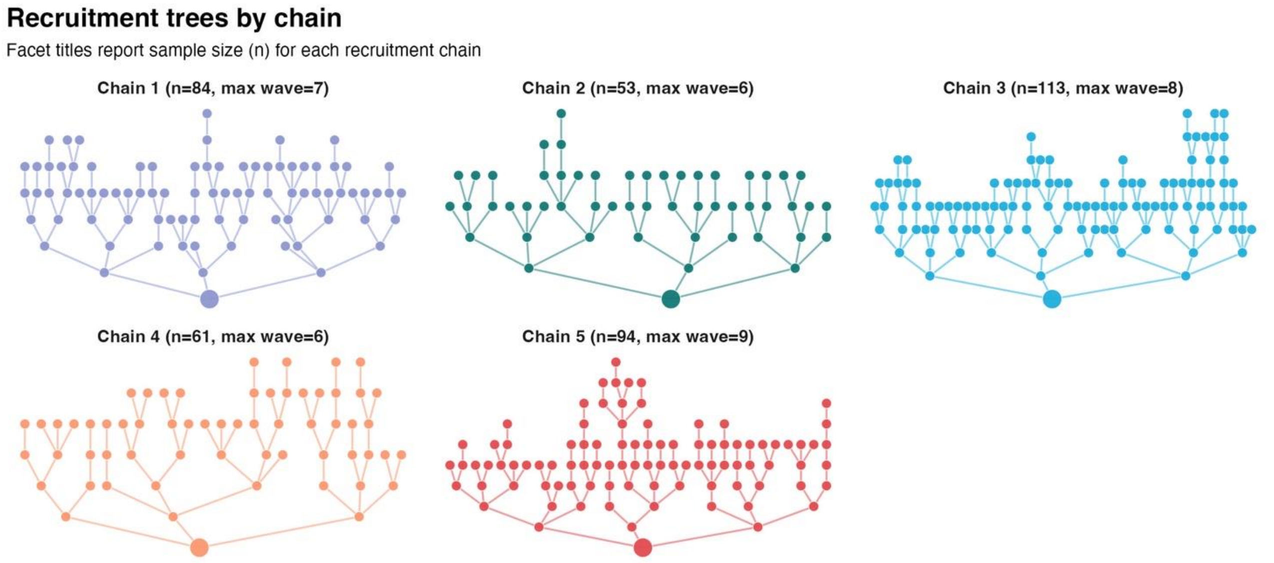

Supplementary Figure S2. RDS Recruitment Chains of Five Seeds (N = 405)

S4. RDS Equilibrium and Degree Diagnostics [5]

To assess the adequacy of the RDS process, we conducted a series of standard diagnostics. Convergence and equilibrium were visually evaluated using RDS-II estimates for four pre-specified key variables (mpox vaccination willingness, age category, sexual orientation, and frequency of condomless anal intercourse in the past year) across increasing recruitment waves. Stabilization of estimates prior to the final recruitment wave was interpreted as evidence that the influence of initial seeds had diminished. One participant reported a network size of zero, which is inconsistent with RDS recruitment assumptions; this observation was excluded from weight computation but retained in unweighted analyses.

The reported personal network size (degree) exhibited a right-skewed distribution, with a median of 15 (IQR: 7-43). Only one participant (1/405) reported a network size of zero. Given that recruitment necessarily implies at least one social connection, this value likely reflects misinterpretation or reporting error rather than a true absence of ties. Due to its negligible frequency, its impact on RDS-II estimates and convergence is unlikely to be material. The distribution of self-reported network size was right-skewed, with most participants reporting moderate network sizes and a small number

reporting very large value (range: 0-2000). On the log scale (**Supplementary Figure S3**), the distribution appeared broadly unimodal with a long right tail, indicating occasional extreme degree reports and little evidence of substantial mass at zero or near-zero values.

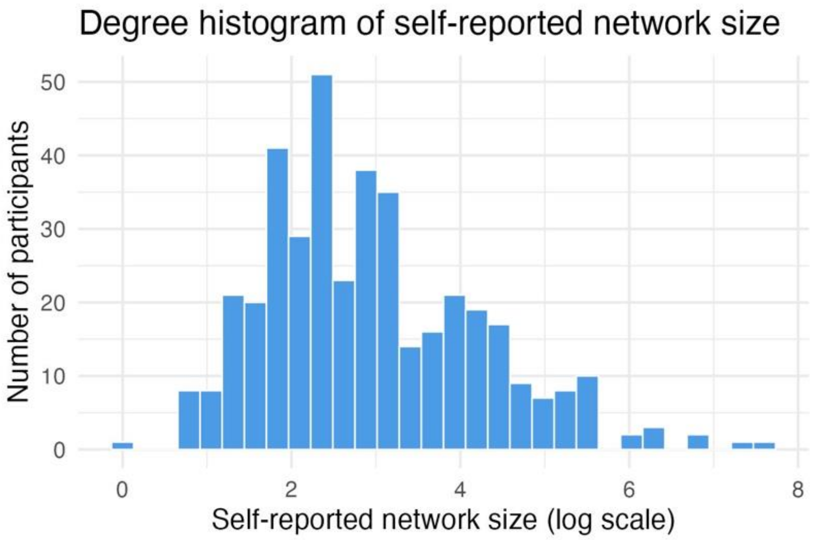

Supplementary Figure S3. The Distribution of Log-transformed Self-reported Network Size (RDS Degree)

Equilibrium diagnostics were conducted for four pre-specified key variables: mpox vaccination willingness, age category, sexual orientation, and frequency of condomless anal intercourse in the past year. Convergence was assessed using RDS-II cumulative estimates plotted against the number of recruited participants. Convergence plots for the key study variables demonstrated stabilization within early recruitment waves, indicating that equilibrium was reached prior to the terminal waves (**Supplementary Figure S4**). These findings collectively support the robustness of the RDS process and reduce concerns regarding seed-dependent bias. These findings support the robustness of the RDS process, reduce concerns regarding seed-dependent bias, and provide additional support for the adequacy of the achieved sample within the sampled network structure.

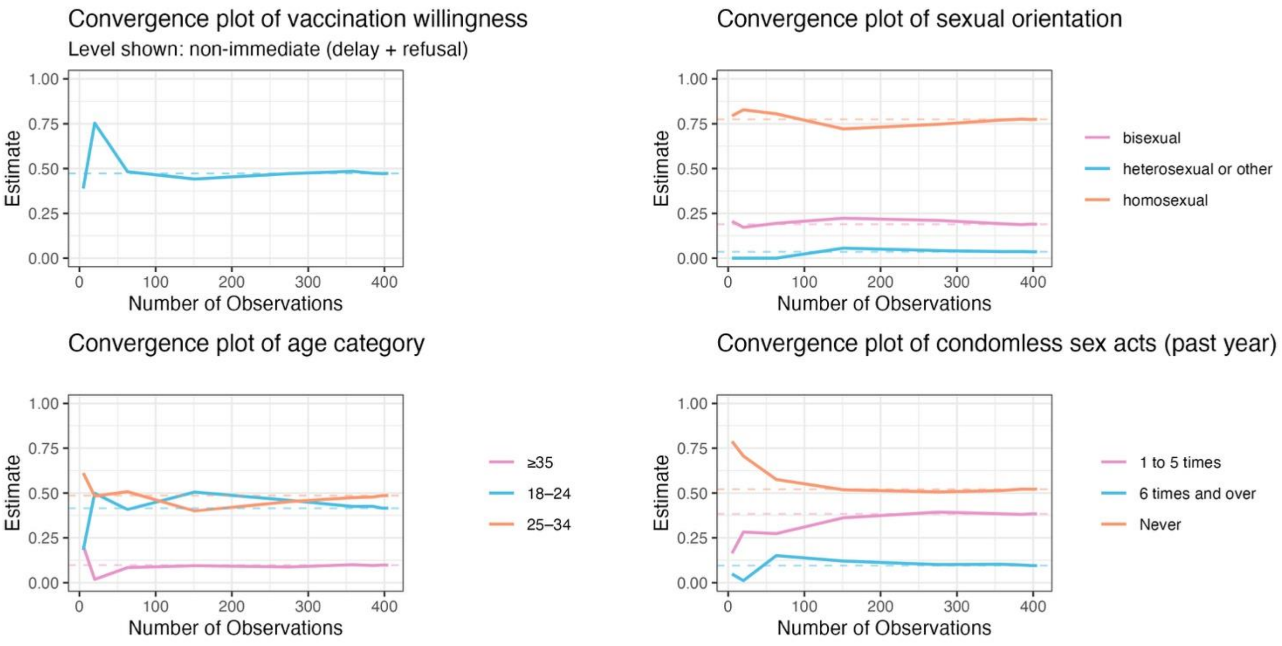

Supplementary Figure S4. Convergence Plots of Four Key Study Variables

S5. RDS Homophily Diagnostics [6]

Recruiter-recruit mixing patterns were examined using cross-tabulation matrices for four pre-specified key variables: mpox vaccination willingness, age category, sexual orientation, and condomless sex frequency in the past year. Rows represent recruiter characteristics and columns represent recruit characteristics. Diagonal cells indicate concordant recruiter–recruit pairs. Higher diagonal concentration suggests homophily, whereas comparable diagonal and off-diagonal frequencies indicate near-random mixing.

Supplementary Table S1 indicated limited to moderate homophily across key variables. Structural attributes such as age, and to a lesser extent sexual orientation, exhibited expected within-group recruitment tendencies, consistent with known social structuring within MSM networks. In contrast, behavioral and attitudinal variables, including condomless sex acts frequency and mpox vaccination willingness, showed near-random mixing, with comparable frequencies of concordant and discordant recruiter-recruit pairs.

- **Sensitivity analyses for the primary outcome**

Because mpox vaccination willingness was the primary outcome, additional analyses were conducted to evaluate potential recruitment-induced dependence. Specifically, recruiter-recruit similarity was evaluated using logistic regression with chain-clustered robust standard errors, and a random-intercept logistic model was fitted to estimate the chain-level intra-class correlation coefficient (ICC).

Logistic regression at the recruiter-recruit dyadic level showed no statistically significant association between recruiter vaccination willingness and recruit vaccination willingness after

accounting for chain-clustered robust standard errors (OR = 1.22, 95% CI: 0.99-1.51, p = 0.068). Furthermore, random-intercept logistic models indicated negligible between-chain variance, yielding an ICC close to zero. These findings provide little evidence that recruitment chains introduced meaningful dependence in the primary outcome.

S6. Mpox Awareness and Psychosocial Constructs

Participants who reported prior awareness of mpox were subsequently administered additional five-point response-scale items across four conceptual domains: (1) **information exposure frequency**, (2) **perceived transmission likelihood**, (3) **perceived risk-group susceptibility**, and (4) **vaccine-related beliefs**. Information exposure items were rated on a five-point frequency scale, whereas items on perceived transmission likelihood, perceived risk-group susceptibility, and vaccine-related beliefs were rated on five-point perceived-likelihood or agreement scales, as appropriate. Higher scores indicated greater frequency of exposure, stronger perceived likelihood, or stronger agreement, depending on the item. Individual item wording and response scales are provided in Supplementary Table S2.

- **Information Exposure Frequency**

Among participants who reported prior awareness of mpox, information exposure was generally low to occasional across channels. Official account posts, short-video platforms, and television/radio/newspapers were most commonly reported at the "occasionally" level, whereas "often" responses were less frequent and "very often" was not selected for any channel. Search engines, MSM online community platforms, MSM offline community venues, and healthcare professionals showed relatively high proportions of "never" responses. Community-based organizations were reported less frequently than major digital channels. Internal consistency (8 items) for this domain was acceptable, with a Cronbach's alpha of 0.794.

- **Perceived Transmission Likelihood**

Perceived transmission likelihood was highest for intimate contact with an infected person, with 79.20% rating this route as likely or very likely. Bites or scratches from infected animals were also frequently endorsed (71.73%). Lower, but still substantial, proportions rated prolonged close exposure to respiratory droplets (55.47%) and contact with contaminated surfaces (51.20%) as likely or very likely. Internal consistency (4 items) for this domain was acceptable, with a Cronbach's alpha of 0.725.

- **Perceived Risk-Group Susceptibility**

Perceived susceptibility varied across population groups. The highest proportions rating groups as likely or very likely to contract mpox were observed for individuals with multiple sexual partners (88.80%), people living with HIV (88.53%), sex workers (84.27%), immunocompromised

individuals (81.33%), and MSM (78.13%). Healthcare workers were much less frequently perceived as likely to contract mpox (25.33%). Internal consistency (6 items) for this domain was acceptable, with a Cronbach's alpha of 0.782.

- **Vaccine-Related Beliefs**

Vaccine-related beliefs were generally favorable. Most participants agreed or strongly agreed that vaccination is an effective way to control mpox transmission (81.87%) and that high-risk groups should be prioritized for vaccination, including individuals with multiple sexual partners (82.40%), sex workers (81.60%), MSM (76.80%), and people living with HIV (73.60%). Agreement was also observed for post-exposure vaccination reducing the likelihood of developing mpox (62.93%) and preventing severe mpox (59.20%). Uncertainty was most pronounced regarding cross-protection from smallpox vaccination, for which 50.40% selected "uncertain." Internal consistency (9 items) for this domain was good, with a Cronbach's alpha of 0.859.

- **Latent Psychosocial Constructs**

Principal component analysis (PCA) was conducted among participants who reported prior awareness of mpox to derive composite psychosocial constructs (see **Supplementary Table S3**). All items were standardized before analysis, and PCA was performed separately within the four conceptual domains: information exposure frequency, perceived transmission likelihood, perceived risk-group susceptibility, and vaccine-related beliefs. The item labeled "Other" (alternative information sources) was excluded from the information exposure frequency domain because of conceptual heterogeneity and low frequency. Specifically, PC1 explained 41.5% of the variance for information exposure frequency, 55.0% for perceived transmission likelihood, 51.5% for perceived risk-group susceptibility, and 48.1% for vaccine-related beliefs. The corresponding standardized alpha values were 0.795, 0.726, 0.801, and 0.860, respectively. These findings supported the use of PC1 as a continuous summary score for each domain. The resulting component scores were retained and incorporated into subsequent multivariable regression models to reduce dimensionality and mitigate multicollinearity.

A conceptual framework was visualized in **Supplementary Figure S5**. Mpox awareness was conceptualized as shaping awareness-conditioned psychosocial dimensions, which may in turn relate to prevention-related behaviors and vaccination willingness, while heterogeneity within the delayed/refused response group was further examined through exploratory concern-profile analysis. As the study was cross-sectional, the figure is intended to summarize conceptual pathways rather than imply confirmed causal direction.

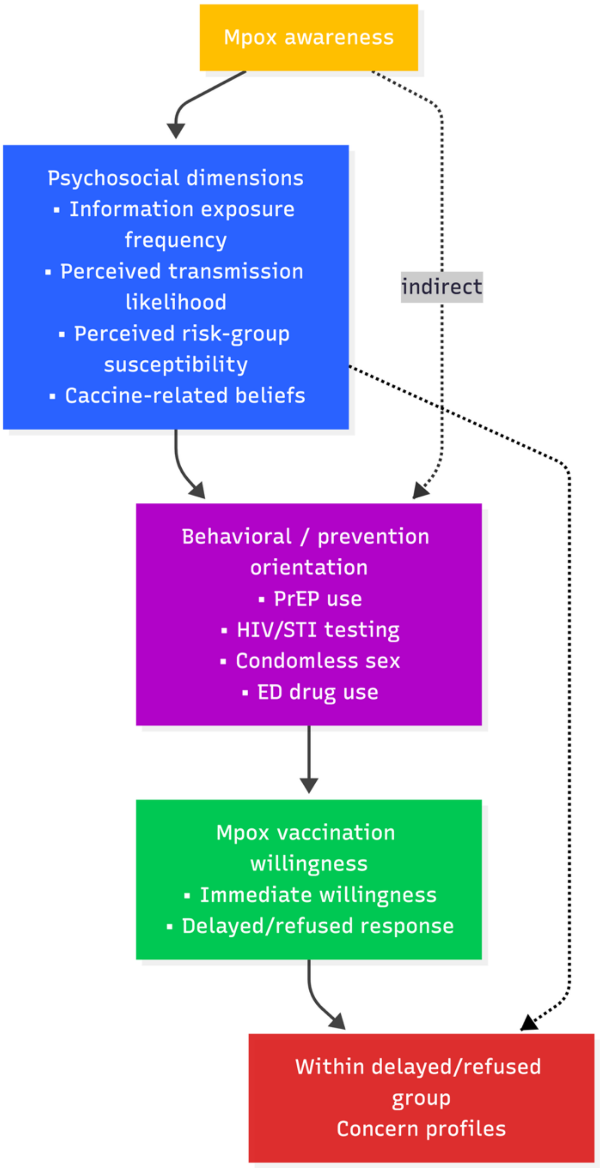

Supplementary Figure S5. Conceptual framework linking mpox awareness, psychosocial dimensions, prevention-related behaviors, and vaccination willingness.

S7. Concern Profiles within the Delayed/Refused Responses Subgroup

To explore heterogeneity within the delayed/refused responses subgroup, analyses were restricted to participants with delayed/refused responses to receive mpox vaccination (n = 204). Concern profiles were derived from responses to eight standardized items assessing vaccination-related concerns, including concerns about vaccine effectiveness, safety, contraindications, privacy related to MSM identity or HIV/STI testing, financial cost, and procedural burden. For descriptive purposes, the proportion of participants who agreed or strongly agreed with each item was calculated within this subgroup. The concern items were measured on five-point agreement scales and are presented in **Supplementary Table S4**.

Unsupervised clustering was applied to these standardized items to identify concern profiles. The open-ended "Other" response was excluded from clustering because of its heterogeneous nature. Clustering was performed using partitioning around medoids (PAM), a medoid-based partitioning method that is relatively robust to outlying response patterns and less sensitive to distributional irregularities than k-means clustering [7]. As shown in the **Supplementary Figure S6**, candidate numbers of clusters were evaluated across k = 2-4 using the average silhouette width together with substantive interpretability [7]. The average silhouette widths were 0.177 for k = 2, 0.162 for k = 3, and 0.156 for k = 4. Although the 2-cluster solution showed the highest internal separation, the 3-cluster solution was retained because it provided a more interpretable balance between internal separation and substantive resolution, whereas the 2-cluster solution collapsed distinct higher-concern patterns and the 4-cluster solution introduced additional fragmentation without clear interpretive gain.

As sensitivity analyses, we compared the retained PAM solution with Ward hierarchical clustering and examined bootstrap-based Jaccard similarity. The hierarchical clustering solution showed broadly similar profile patterns to the retained 3-cluster PAM solution (**Supplementary Table S5**). In particular, both approaches identified (i) a broadly elevated multi-concern profile, (ii) a lower-concern / low-urgency profile, and (iii) a more selective safety- and burden-related concern profile, although the allocation of observations across clusters differed somewhat between algorithms. Bootstrap-based resampling suggested moderate stability overall (mean Jaccard = 0.785; median = 0.828; mean minimum cluster Jaccard = 0.712).Cluster profiles were summarized by the mean item score (range: 1-5) for each concern item within each cluster, with higher values indicating stronger endorsement of the stated concern. Cluster labels were assigned post hoc according to dominant response patterns and were used for descriptive interpretation only.

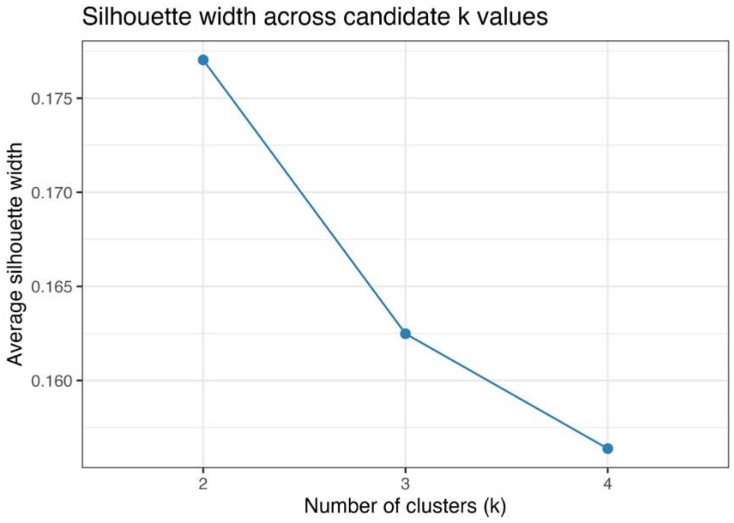

Supplementary Figure S6. Average Silhouette Width across Candidate PAM Solutions (k = 2-4) for the Delayed/Refused Responses Subgroup.

S8. Modelling Strategy Overview and Supplementary Technical Details

A schematic overview of the modelling strategy is provided in **Supplementary Figure S7**. This figure summarizes the study sample, primary outcome definition, variable construction, primary inferential model, complementary robustness analyses, and exploratory subgroup analyses. For Model 1 (standard logistic regression with chain-clustered robust standard errors), the smaller outcome category contained 201 participants, and the model included 15 free parameters, yielding an events-per-variable (EPV) ratio of 13.4. This indicates that the primary inferential model was not highly parameterized relative to the available sample size.

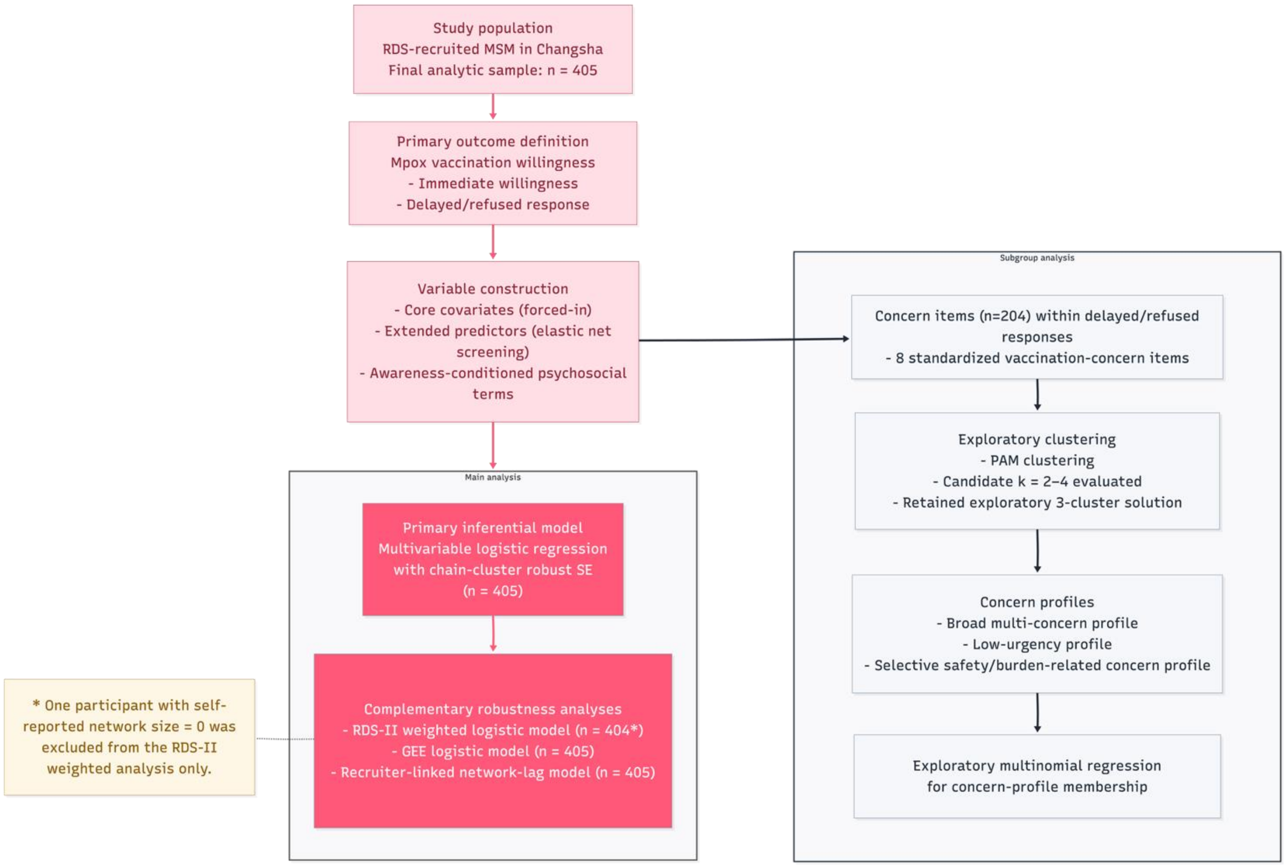

Supplementary Figure S7. Schematic overview of the analytic strategy. Cluster-robust logistic regression was treated as the primary inferential model. The RDS-II weighted, GEE, and recruiter-linked network-lag models were used as complementary robustness analyses. Concern-profile clustering and multinomial regression within the delayed/refused response subgroup were exploratory. One participant with self-reported network size = 0 was excluded from the RDS-II weighted analysis only.

Covariate Preparation

A separate degree variable was constructed for RDS weighting procedures. Recruitment chain identifiers were retained to account for within-chain correlation in clustered analyses. Demographic and behavioral variables were harmonized prior to modeling. Categorical variables were recoded as factors with pre-specified reference levels. Network size measures (total MSM acquaintances,

online acquaintances, and sexual partners) were treated as numeric and log-transformed to reduce right-skewness and mitigate the influence of extreme values.

Technical Details of Penalized Screening

For penalized screening, a design matrix was constructed using the extended predictors only, and the full analytic sample was used for model fitting. Elastic net models were fit using 10-fold cross-validation with binomial likelihood and deviance loss. The tuning parameter was selected using the minimum cross-validated deviance criterion (lambda.min), and predictors with non-zero coefficients at the selected penalty were retained. The final multivariable regression specification combined the forced-in core covariates with the penalization-selected predictors.

S9. Recoded Variables in Multinomial Regression for Concern-Profile Membership

Because several categories were sparse within the delayed/refused responses subgroup (notably heterosexual or other sexual orientation and HIV-positive status), selected variables were recoded prior to modeling to reduce instability and quasi-complete separation. Sexual orientation was recoded as homosexual versus non-homosexual (combining bisexual and heterosexual/other). HIV-related behavioral status was recoded into three categories: HIV negative without testing in the past three months (no_recent_test), HIV negative with testing in the past three months (recent_test), and a combined sparse category (positive_or_prefer_not_to_say) grouping HIV-positive participants with those who preferred not to disclose their HIV status. This collapsing strategy was implemented to stabilize model estimation while retaining participants with rare or undisclosed HIV-status responses. Full results are presented in Supplementary Table S7.

References

1. Salganik, M.J., *Variance Estimation, Design Effects, and Sample Size Calculations for Respondent-Driven Sampling.* Journal of Urban Health, 2006. 83(1): p. 98-112.
2. Gile, K.J. and M.S. Handcock, *Respondent-Driven Sampling: An Assessment of Current Methodology.* Sociol Methodol, 2010. 40(1): p. 285-327.
3. Gile, K.J., L.G. Johnston, and M.J. Salganik, *Diagnostics for Respondent-Driven Sampling.* Journal of the Royal Statistical Society Series A: Statistics in Society, 2015. 178(1): p. 241-269.
4. Heckathorn, D.D., *Respondent-Driven Sampling: A New Approach to the Study of Hidden Populations*.* Social Problems, 1997. 44(2): p. 174-199.
5. Handcock, M.S., K.J. Gile, and C.M. Mar, *Estimating hidden population size using Respondent-Driven Sampling data.* Electron J Stat, 2014. 8(1): p. 1491-1521.
6. Salganik, M.J. and D.D. Heckathorn, *Sampling and Estimation in Hidden Populations Using Respondent-Driven Sampling.* Sociological Methodology, 2004. 34(1): p. 193-240.
7. Kaufman, L. and P. Rousseeuw, *Finding Groups in Data: An Introduction To Cluster Analysis*. 1990.
